# Gut Microbiome and Mycobiome Alterations in an In Vivo Model of Alzheimer’s Disease

**DOI:** 10.3390/genes13091564

**Published:** 2022-08-31

**Authors:** Valeria D’Argenio, Iolanda Veneruso, Chunmei Gong, Valentina Cecarini, Laura Bonfili, Anna Maria Eleuteri

**Affiliations:** 1 Department of Human Sciences and Quality of Life Promotion, San Raffaele Open University, Via di Val Cannuta 247, 00166 Roma, Italy; 2 CEINGE-Biotecnologie Avanzate, Via G. Salvatore 486, 80145 Napoli, Italy; 3 Department of Molecular Medicine and Medical Biotechnologies, Federico II University, Via Sergio Pansini 5, 80131 Napoli, Italy; 4 School of Biosciences and Veterinary Medicine, University of Camerino, Via Gentile III da Varano, 62032 Camerino, Italy

**Keywords:** Alzheimer’s disease, microbiome, mycobiome, metagenomic studies, 3xTg-AD mice

## Abstract

Gut microbiota has emerged as an important key regulator of health and disease status. Indeed, gut microbial dysbiosis has been identified in an increasing number of diseases, including neurodegenerative disorders. Accordingly, microbial alterations have been reported also in Alzheimer’s disease (AD), suggesting possible pathogenetic mechanisms contributing to the development of specific AD hallmarks and exacerbating metabolic alterations and neuroinflammation. The identification of these mechanisms is crucial to develop novel, targeted therapies and identify potential biomarkers for diagnostic purposes. Thus, the possibility to have AD in vivo models to study this microbial ecosystem represents a great opportunity for translational applications. Here, we characterized both gut microbiome and mycobiome of 3xTg-AD mice, one of the most widely used AD models, to identify specific microbial alterations with respect to the wild-type counterpart. Interestingly, we found a significant reduction of the *Coprococcus* and an increased abundance of *Escherichia_Shigella* and *Barnesiella* genera in the AD mice compatible with a pro-inflammatory status and the development of AD-related pathogenetic features. Moreover, the fungal *Dipodascaceae* family was significantly increased, thus suggesting a possible contribution to the metabolic alterations found in AD. Our data point out the strict connection between bacterial dysbiosis and AD and, even if further studies are required to clarify the underlining mechanisms, it clearly indicates the need for extensive metagenomic studies over the bacterial counterpart.

## 1. Introduction

It has been well established that the gut microbiota plays an important role in health status acquisition and maintenance, and, consequently, its alterations can be associated with several diseases [1]. This statement is true not only for intestinal diseases but can be extended also to extra-intestinal disorders [1]. In particular, it is well known that a stable and complex communication system exists between gut and brain, and the gut microbiota has been claimed as a factor able to influence the gut–brain axis [2,3,4]. As a consequence, it has been proposed that a microbial dysbiosis at the gut level, by acting through different mechanisms such as membrane permeability modifications, inflammatory response induction or toxic metabolite production, may play a role also in neurodegenerative disorders such as Alzheimer’s disease (AD) [5]. Accordingly, several studies have shown a correlation between gut microbiota alterations and cognitive impairment and/or specific AD features [5,6,7]. These studies, even if often observatory and at a preliminary stage, have the overall merit of having highlighted an additional molecular mechanism involved in AD pathogenesis that has the potential advantage to be easily actionable for prevention or therapeutic purposes [8]. In this context, it has been reported that a high-fat diet is able to reduce the *Lactobacillus*, *Bacteroides* and *Prevotella* species and increases *Bifidobacterium* abundance in mice. Interestingly, this microbial dysbiosis is related to alterations of gut permeability, as assessed by zonulin and occludin proteins reduced expression, and to a consequent increase of circulating bacterial lipopolysaccharide (LPS) and other pro-inflammatory markers [9]. More recently, a high-fat diet was reported to induce metabolic alterations, cognitive impairment and tau hyperphosphorylation in transgenic mice [10]. These findings suggest that lifestyle changes (i.e., dietary modifications), by modifying the gut microbiota composition, may ameliorate some AD typical features. Indeed, probiotics administration has shown promising results. Kaur et al. administered a 2-month probiotic supplementation to an AD mouse model and reported an improvement of gut functions and reduced anxiety-like behavior [11]. Webberley et al. tested a probiotic consortium in 3xTg-AD mice and reported improved cognitive functions, reduced systemic inflammation and a lower Firmicutes:Bacteroidetes ratio in the treated group [12]. 

Using the same AD model (3xTg-AD mice), we reported that the oral administration of a lactic acid bacteria and *Bifidobacteria* formulation was able to reduce cognitive decline and Aβ aggregates deposition ameliorating also neuronal proteolytic pathways [13]. Moreover, the same formulation was found to reduce the oxidative stress by a sirtuin-1 (SIRT1)-dependent mechanism [14], to ameliorate glucose homeostasis and decrease phosphorylated tau aggregates [15], and to positively impact lipid metabolism [16]. Finally, we have recently evaluated the effects of a yeast-enriched beer on the gut microbiota and neurodegeneration of 3xTg-AD mice, showing a positive modulation in the treated group [17]. Since 3xTg-AD mice are a common model used for studying AD related-features and the underlying mechanisms, including gut microbiota modifications, the definition of the microbial differences between 3xTg-AD and wild-type (WT) mice may be important to design more targeted studies. Here, we report the bacterial and fungal microbiota composition of 3xTg-AD mice, showing specific features with respect to the WT group that will be useful for further investigations aimed to clarify their role in AD pathogenesis.

## 2. Materials and Methods

### 2.1. Animals and Study Design

AD triple-transgenic mice, B6;129-Psen1tm1Mpm Tg (APPSwe, tauP301L) 1Lfa/J (named 3xTg-AD) and the wild-type B6129SF2 mice were purchased from the Jackson Laboratory (Bar Harbor, ME, USA). 3xTg-AD mice contain three mutations associated with frontotemporal dementia or familial AD (amyloid precursor protein [APP] Swe, tau MAPT P301L and presenilin-1 M146V). This reliable model of human AD displays both plaque and tangle pathology, with Aβ intracellular immunoreactivity detectable at three months of age and hyperphosphorylation of tau protein occurring by 12 to 15 months of age [18], reliably reproducing traits similar to those observed in the entire life of Alzheimer’s disease patients.

Experiments complied with the ARRIVE guidelines, in accordance with the EU Directive 2010/63/EU for animal experiments, and with a protocol approved by the Italian Ministry of Health (518/2018-PR). Mice were housed in plastic cages (Makrolon, Covestro A.G., Filago, Italy) in a temperature-controlled room (21 ± 5 °C) and 60% humidity on 12-hour light/dark reversed cycle (light was switched on at 8:00 p.m.) and maintained on standard laboratory diet (Mucedola, Settimo Milanese (MI), Italy) and water ad libitum. Appropriate measures minimized pain and discomfort in experimental animals.

### 2.2. Fecal Samples Collection and DNA Extraction

Eleven 8-week-old mice, 5 AD and 6 WT mice, were analyzed. Fecal samples were collected from each mouse at the time of sacrifice and were immediately frozen at −80 °C until further processing. Genomic DNA was extracted from all stool samples by using the RSC Blood DNA kit and the Maxwell RSC instrument (both from Promega, Madison, WI, USA), as previously reported [19]. Specifically, 100 mg of each stool sample were lysed by adding 400 µL of Lysis Buffer, pestled and vortexed until reached a homogeneous status and incubated at 95 °C for 5 min at 800 rpm on thermomixer. A centrifugation step of 5 min at 13,000 rpm was carried out and 300 µL of the supernatant were transferred into a fresh tube. Thirty µL of Proteinase K solution were added to the samples that were vortexed and incubated at 56 °C for 20 min at 500 rpm on thermomixer. Next, the lysed samples were loaded onto the RSC cartridge and processed from the Maxwell RSC instrument. Final DNA samples were eluted in 100 µL of Elution Buffer and quantitatively assessed by using the Nanodrop spectrophotometer (Thermo Fisher Scientific, Waltham, MA, USA).

### 2.3. Bacterial 16S rRNA Analysis

Fecal bacterial community analysis was carried out using 16S rRNA custom primers designed to selectively amplify the V4-V6 hypervariable regions, as previously described [20]. PCR mix solutions and amplification conditions were optimized to guarantee the correct target amplification. In particular, AmpliTaq Gold polymerase, GC enhancer (both from Thermo Fisher Scientific, Waltham, MA, USA) and 20 µM of forward and reverse custom primers were used. After 2% agarose gel electrophoretic analysis, all the PCR products were purified by using the AMPure XP beads (Beckman Coulter, Brea, CA, USA) and quality-checked on the Tape Station System with the D1000 ScreenTapes (both from Agilent Technologies, Santa Clara, CA, USA). Next, the purified amplicons were quantified with Qubit HS (Qubit, dsDNA HS Assay, Life Technologies, Carlsbad, CA, USA) and diluted to 2 ng/µL in order to being processed with a second-round PCR, necessary to add specific indexes to each sample and also the universal adapters for the following NGS reactions. During this step, Nextera DNA CD Indexes (Illumina, San Diego, CA, USA) were used. Then, further AMpure XP beads-based purification and Tape Station qualitative analysis were carried out. Finally, 5 µL of each library were pooled to be sequenced in a single sequencing run. A total of six blank samples (2 for DNA extraction, 2 for the first PCR and 2 for the second PCR) was processed together with the others to check for any contaminants and environmental biases potentially occurring during the experimental procedure.

Sequencing reaction was carried out using the MiSeq reagent Kit V3 600 cycles on the MiSeq instrument (Illumina, San Diego, CA, USA). The library’s pool was loaded at a final concentration of 9 pM with a 30% PhiX.

### 2.4. Fungal ITS Analysis

Mycobiome analysis of fecal communities was carried out on the same samples to assess also fungal composition. Specific custom primers for ITS1 amplification were used as previously reported [17]. After this first-round PCR and a beads-based purification step (AMPure XP beads, Beckman Coulter, Brea, CA, USA), the amplicons were quality assessed by using a D1000 ScreenTape (Agilent Technologies, Santa Clara, CA, USA) and further amplified to allow samples indication and add the sequencing adapters. The obtained libraries were then purified and visualized on the Tape Station (Agilent Technologies, Santa Clara, CA, USA) before being pooled for sequencing. Blank samples corresponding to DNA extraction and the 2 PCR rounds (totally, *n* = 6) were also processed to avoid environmental contaminants. Next-generation sequencing was performed on the Illumina MiSeq system (V3, 300 × 2 PE) with the same conditions specified above.

### 2.5. Bioinformatics Analysis of Metagenomic Data

The FASTQ files generated by the sequencing run were processed by the CRG bioinformatic facility (https://biocore.crg.eu/wiki/Main_Page, last accession on 1 July 2022). In particular, sequencing reads quality was assessed by FastQC [21] and primary data analysis was carried out using the mothur tool (version 1.44.1) [22], according to its pipeline (https://mothur.org/wiki/miseq_sop/, last accession on 1 July 2022). Reference sequences for reads mapping were obtained (i) from the SILVA database, version 138 [23] for the bacterial 16S rRNA, and (ii) from the UNITE database, version 04.02.2020 [24] for the ITS. Next, the R packages Phyloseq v. 1.30.0 [25] and microbiome v. 1.8.0 [26] were used to estimate α- and β-diversity [27], while the identification of significantly enriched taxa in the 2 study groups, was evaluated by using the R package DESeq2 v. 1.26.0 [28]. 

The mothur output was further analyzed using the Microbiome Analyst tool [29]. In particular, samples richness and/or evenness were evaluated using the ANOVA test to assess any significant difference. β diversity was measured using the unweighted and weighted UniFrac distance measures coupled with the PERMANOVA test to verify the significance of samples grouping. Linear discriminant analysis (LDA) effect size (LEfSe) was used to detect significant differentially abundant taxa based on the Kruskal–Wallis rank sum test coupled with linear discriminant analysis to assess the relevance of any differential abundant taxa (*p*-value cutoff: 0.1, FDR adjusted. Log LDA score: 2).

## 3. Results

### 3.1. Microbiome Composition Analysis

All the collected fecal samples were analyzed as described under the Methods section to assess their bacterial composition and verify any significant feature able to discriminate between the two studied groups. An average of 29,030 reads/sample, corresponding to a total of 126 operational taxonomic units (OTUs), was obtained and used for subsequent analyses. It has to be underlined that the six negative samples, used as analytic controls for the experimental procedure, did not produce reads, assessing the absence of contaminants during samples preparation and were not further considered for the downstream analyses.

**Figure 1:**
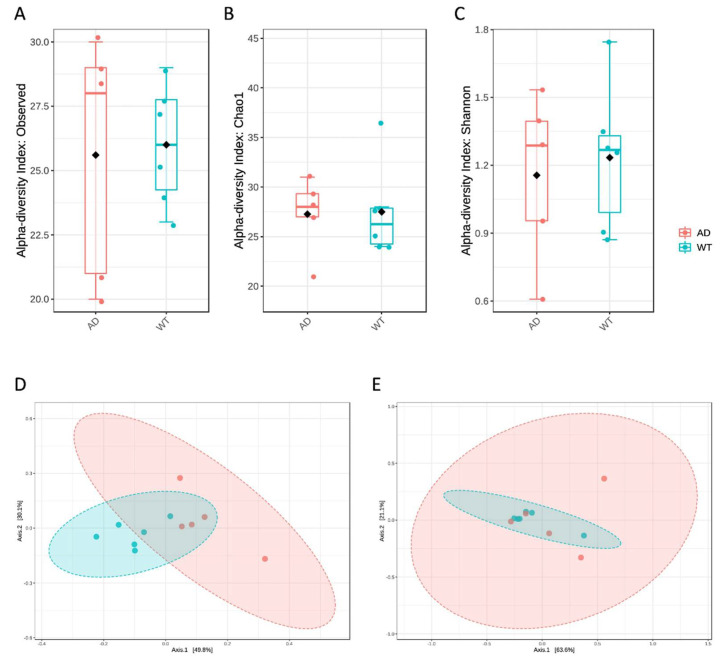
Diversity measures of the bacterial taxa identified in the AD and WT mice. Alpha diversity was calculated using several metrics to measure the within-group diversity and bacterial richness. In particular, observed species (**A**), Chao1 (**B**) and Shannon (**C**) indices were obtained showing no significant differences between the 2 tested conditions. β diversity was also measured using the unweighted (**D**) and weighted (**E**) UniFrac distance measures. Statistical significance was assessed by PERMANOVA test, resulting in significance (*p* < 0.005) only in the case of unweighted UniFrac.

First, diversity measures were obtained to evaluate both the within- and between-group variabilities (Figure 1). In particular, both richness (Figure 1A,B) and evenness (Figure 1C) did not highlight significant differences between the tested conditions, also using different metrics. However, β diversity, as obtained by unweighted UniFrac distance measure, showed a significantly different microbial composition between AD and WT mice (*p* < 0.005, Figure 1D), even if not confirmed by using the weighted UniFrac distance metric (Figure 1E). As already reported elsewhere [17], unweighted and weighted UniFrac measures are respectively a quality-based and a quantity-based index. Thus, the result reported herein may indicate that the variation between the two compared groups is not due to the taxa abundances but rather to the type of taxa present in the microbiome.

**Figure 2:**
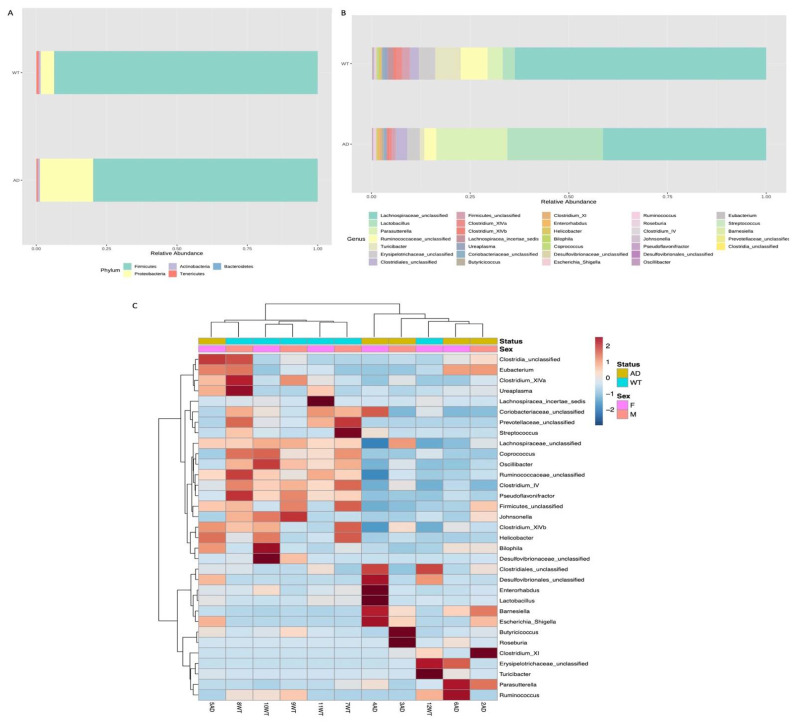
Bacterial composition of AD and WT mice gut microbiome. Taxonomic assignment is shown at phylum (**A**) and genus (**B**) level. For each of these taxonomic levels, the barplots show the relative abundance (%) of each identified taxon/group. A different bacterial composition is highlighted both at phylum and at genus level. A heatmap of variance was obtained by grouping the reads based on the assigned taxa: a good cluster was obtained at genus level showing a clear separation between AD and WT mice (**C**).

Taxonomic assignment highlighted the presence of a total of five phyla, Firmicutes and Proteobacteria being the most abundant in both groups. However, we observed a reduction of Firmicutes (from 93.8% to 79.8%) and an increase of Proteobacteria (from 4.7% to 18.8%) relative abundances in the AD respect to the WT mice (Figure 2A). 

**Figure 3:**
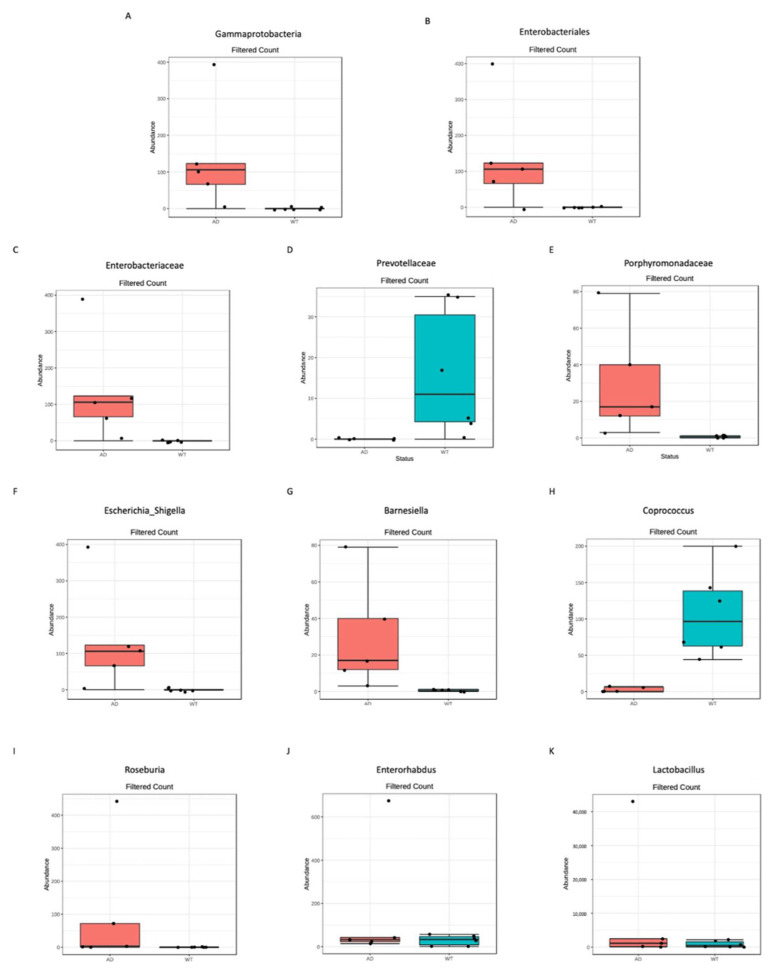
Significantly different taxa identified between AD and WT mice. Differential abundance analysis was performed to highlight significantly different taxa using DESeq2, *p*-value < 0.05 after FDR correction; 1 class (**A**), 1 order (**B**), 3 families (**C**–**E**), and 6 genera (**F**–**K**) resulted in significant differences between the 2 tested conditions.

At the genus level, we found in the AD mice an increased abundance of *Lactobacillus* (from 0.3% to 24%) and *Parasutterella* (from 3.8% to 17.9%) and a reduced abundance of *Lachnospiraceae_unclassified* (from 63.7% to 41.3%), *Ruminococcaceae_unclassified* (from 6.8% to 3%) and *Turicibacter* (from 6.4% to 1%) genera (Figure 2B). Next, a clustering analysis was carried out to verify if these observed different taxonomic abundances are able to cluster the analyzed samples: as shown in Figure 2C, at the genus level, samples from the same group are more similar each to the others than samples from the other group, highlighting also differential abundance patterns between AD and WT mice. Thus, to verify the presence of statistically significant differences between the two study groups differential abundance analysis was performed. No significant taxa were identified at the phylum level; however, we found one class, one order, three families and six genera significantly different between AD and WT mice (Figure 3).

In particular, among the differentially abundant taxa, the *Gammaproteobateria* class (Figure 3A), the *Enterobacteriales* order (belonging to *Gammaproteobateria* class, Figure 3B), the *Enterobacteriaceae* family (belonging to *Enterobacteriales* order, Figure 3C) and the *Escherichia_Shigella* genus (belonging to *Enterobacteriaceae* family, Figure 3F) were significantly more abundant in the AD respect to the WT mice. Moreover, other two families, both belonging to the Bacteroides phylum, were significantly different, with the *Prevotellaceae* family being more abundant in the WT mice (Figure 3D) and the *Porphiromonadaceae* family in the AD (Figure 3E). Finally, at the genus level, the other five genera were significantly different between the two study groups: the *Barnesiella* (belonging to Bacteroides phylum), the *Roseburia* and the *Lactobacillus* (both belonging to Firmicutes phylum) and the *Enterorhabdus* (belonging to Actinobacteria phylum) genera being more abundant in the AD mice, while the *Coprococcus* genus (belonging to Firmicutes phylum) was less abundant in the AD mice with respect to the WT.

Finally, linear discriminant analysis (LDA) effect size (LEfSe) showed that at the genus level two taxa are most likely to explain the differences between the two study groups, i.e., the *Barnesiella* and the *Coprococcus* genera, respectively, were more abundant in the AD and in the WT mice.

### 3.2. Mycobiome Composition Analysis

The fungal contribution to gut microbiota was also investigated in the same study groups. A total of 469 OTUs was identified with and average reads/sample of 95,194.

**Figure 4:**
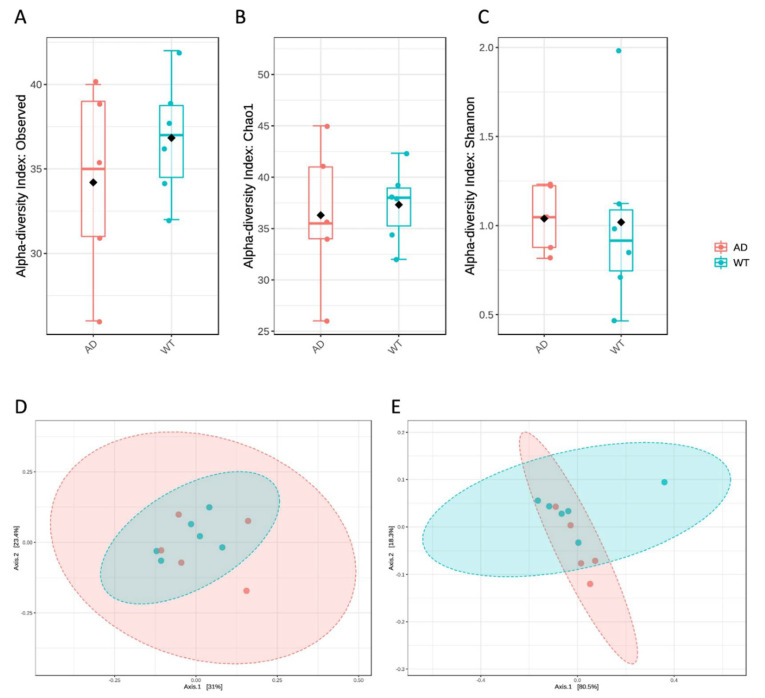
Diversity measures of the fungal taxa identified in the AD and WT mice. α diversity was calculated using observed species (**A**), Chao1 (**B**) and Shannon (**C**) indices. No significant differences were highlighted between the 2 tested conditions. β diversity was also measured using the unweighted (**D**) and weighted (**E**) UniFrac distance measures; in this case also, no significant differences were observed.

Diversity indices were measured showing no significant differences between AD and WT mice (Figure 4), even if it is possible to observe a trend in the reduction of richness in the AD mice (Figure 4A,B), while evenness seems to increase (Figure 4C).

**Figure 5:**
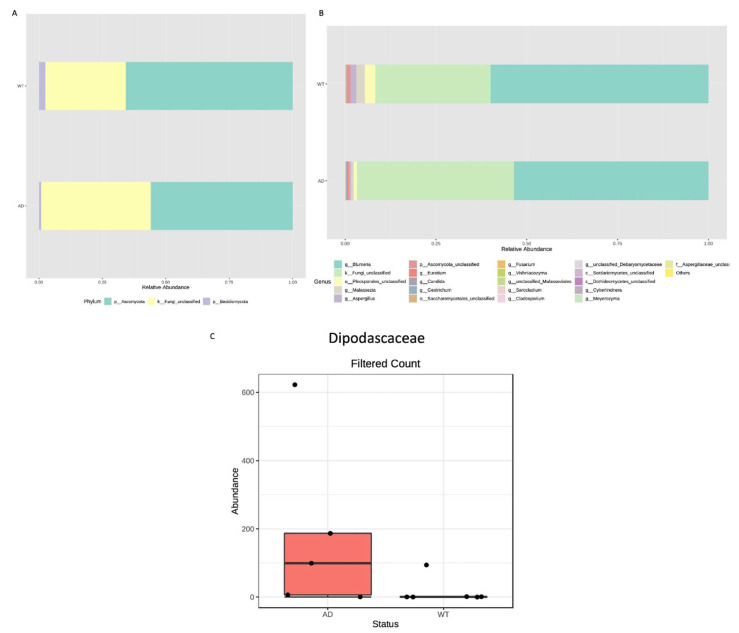
Fungal microbiota composition of AD and WT mice. Taxonomic assignment is shown at phylum (**A**) and genus (**B**) levels as relative abundance (%) of each identified taxon/group. Differential abundance analysis highlighted just one significantly different taxon, the *Dipodascaceae* family, which appears increased in the AD compared with the WT mice (**C**).

Taxonomic assignment allowed the identification of two phyla, Basidiomycota and Ascomycota, both being more abundant in the WT respect to the AD mice (Figure 5A). A different fungal composition is highlighted also in the other taxonomic ranks up to the genus level (Figure 5B). Thus, differential abundance analysis was performed to verify any significant difference. Only a family resulted in significant differences (*p* = 0.001): the *Dipodascaceae*, which appears more abundant in the AD group (Figure 5C).

## 4. Discussion

Gut microbiota has emerged as an important factor contributing to AD pathogenesis [30,31]. This observation has prompted the research in the field due to the potential of using microbiota features for diagnostic purposes and, mainly, as actionable targets of specific therapies. In this context, the use of in vivo models resembling AD has shown its efficacy in evaluating the effects of different therapies on cognitive functions and AD pathogenesis. Here, we describe the bacterial and fungal gut microbiota content of 3xTg-AD mice with respect to WT mice in order to verify the presence of specific signatures related to the disease status. Interestingly, our data suggest that microbiota alterations are mainly related to specific taxa rather than quantitative differences. This is highlighted not only by diversity indices but also by clustering analysis showing bacterial communities more similar within- than between-group variation. Taxonomic analysis showed a reduction of Firmicutes phylum in the AD mice. Even if this difference was not statistically significant, the finding is also in line with previous report in humans [7]. In particular, the reduction of the Firmicutes phylum has been described in several diseases, including diabetes and obesity [32,33]. Since glucose metabolism alterations, such as insulin resistance, have been associated to an increased risk of developing AD, it has been hypothesized that gut dysbiosis may promote AD progression by inducing insulin resistance [7]. Interestingly, in our study, the *Coprococcus* genus, within the Firmicutes phylum, was significantly less abundant in the AD respect to the WT mice and this was one of the two bacterial features able to explain the difference between the two compared groups. *Coprococcus* is usually considered as an anti-inflammatory bacterium, and its abundance has been recently reported as reduced in seven different brain-related diseases, including AD (specifically, attention deficit hyperactivity disorder, autism spectrum disorder, schizophrenia, Alzheimer’s disease, Parkinson’s disease, major depressive disorder and bipolar disorder) [34]. In addition, the *Coprococcus* genus is involved in carbohydrate fermentation and short-chain fatty acid (SCFAs) production [35]. A reduction of SCFAs production is able to induce inflammation, leaky gut and microglial activation and has been consequently related to the pathogenesis of human diseases [31,36,37] Thus, the *Coprococcus* reduction we observed in the AD mice may play a role in disease-related dysbiosis and in the development of the typical pathogenetic clues.

On the contrary, we highlighted an increase of the phylum Proteobacteria in AD respect to WT mice with a significant increase, at the genus level, of the *Escherichia_Shigella* taxon. Similarly, we also reported significant changes of taxa belonging to the Bacteroidetes phylum including, in the AD mice, the increase of the *Barnesiella* genus. These genera, as the other members of both Proteobacteria and Bacteroidetes phyla, are Gram-negative bacteria having the lipopolysaccharide (LPS) as a major component of their outer membrane. LPS proinflammatory properties have been well established [38], and, accordingly, the increase of Bacteroidetes and/or Proteobacteria phyla has been found in different diseases, including AD [7,32,39]. It has to be mentioned that LPS has been reported as a factor able to promote amyloid deposition and tau-related pathology [40,41]. Moreover, LPS and *Escherichia coli (E. coli)* fragments have been found in post-mortem brain tissues from AD patients as co-localizers of amyloid plaques [42]. *E. coli* has been also reported as one of the bacterial strains able to produce amyloid [31,43]. This bacterial amyloid is able to trigger pro-inflammatory responses by recognizing the TLRs and by promoting additional amyloid and misfolded Aβ production, leaky gut and neuroinflammation [43,44,45]. Considering all the above, the significant increase of such Gram-negative genera we found in the gut microbiota of AD mice is compatible with increased LPS translocation into the blood and with pro-inflammatory activities able to promote AD.

In addition to bacterial content, we also investigated fungal fecal composition. Diversity analysis showed a reduction, even if not statistically significant, of fungal richness in AD respect to WT mice. Taxonomic assignment highlighted a different composition and just one significantly different taxon, i.e., the *Dipodascaceae* family, which is most represented in AD mice. Members of the *Dipodascaceae* family have been reported as potential colonizers of the human gut, even if their role is still to be clarified [46]. A study analyzing the gut mycobiome of obese subjects reported the increase of the *Dipodascaceae* family in the obese group and found also a positive correlation with adiposity, serum total cholesterol and fasting triglycerides [47]. Even if further studies are required to confirm this finding, since both glucose and lipids metabolisms are impaired in AD, it is conceivable to suppose the involvement of the *Dipodascaceae* family in these processes.

## 5. Conclusions

Taken together, our data show the presence of a different microbiome and mycobiome composition in 3xTg-AD respect to WT mice. In particular, significantly different taxa potentially contributing to AD pathogenetic clues were identified. Even if further studies are required to move towards a more mechanistic interpretation, our results suggest possible key microbial taxa of AD-related dysbiosis potentially contributing to the disease. In addition, most metagenomic studies to date focus just on bacterial content, while gut communities are more complex ecosystems. Here, we studied also the fungal composition highlighting a significantly different abundance of the *Dipodascaceae* family. This reinforces the hypothesis that several microbial alterations participate to metabolic and pro-inflammatory imbalances that, consequently, can enhance the development of pathological conditions. Finally, since the microbial alterations found in the 3xTg-AD model resemble those found also in humans, we assessed it as an effective model to study AD microbiome and to test possible therapeutic interventions based on microbiota manipulation.

## Data Availability

Data are contained within the article.
